# Supplementary material for: Applying the open-LUCIS framework to identify and characterize human–wildlife conflicts: A case study in Botswana
Source: PLoS One. 2026 Jan 23;21(1):e0336326. doi: 10.1371/journal.pone.0336326 (PMC12829932; doi:10.1371/journal.pone.0336326)
Supplement: Attachment — Submitted filename: Plos one.docx [file pone.0336326.s001.docx]

**Title: Identifying and Characterizing Human-Wildlife Conflicts Using an Open-Source Suitability-Based Framework: A Case Study in Botswana.**

1. **Review of the Introduction Section**

**Strengths**

- The introduction effectively sets the stage by highlighting the global scale of HWC(e.g., Kenya, India, China) and its socio-economic impacts (crop loss, fatalities).
- Strong emphasis on policy responses (e.g., KAZA Transfrontier Conservation Area, UNDP initiatives) to demonstrate real-world relevance.
- Critiques shortcomings of current mitigation strategies (e.g., electric fencing, tech-based alerts) and argues for land-use planning (LUP) as a systemic solution.
- Clearly positions Open-LUCIS as an improvement over proprietary LUCIS by being open-source, participatory, and reproducible.
- Introduces sensitivity analysis as an innovative addition to assess factor influence on HWC.
- Progresses smoothly from global → national → local perspectives, culminating in the study’s specific objectives.

**Weaknesses and Concerns**

- While global cases (Kenya, India) are useful, the link to Botswana’s Chobe District (study area) is underdeveloped. A paragraph contrasting global trends with local Chobe dynamics (e.g., elephant conflicts, agricultural expansion) would strengthen focus.
- Claims LUCIS is "effective" but cites UNDP procurement notices (41,42) as evidence rather than peer-reviewed outcomes. Needs specific examples of LUCIS reducing HWC (e.g., success metrics from Botswana’s Tawana/Chobe).
- Open-LUCIS’s limitations (e.g., computational demands, stakeholder bias in weighting) are not addressed.
- Lacks discussion of competing land-use frameworks (e.g., Marxan, InVEST) to justify why LUCIS is superior for HWC.
- No mention of spatial ecology theories (e.g., landscape permeability, source-sink dynamics) that ground habitat-suitability modeling.
- Mentions "domain knowledge" and "local priorities" but doesn’t detail how stakeholders were involved (e.g., workshops, surveys) or how their inputs were weighted.

2. **Review of the Material and methods Section**

**Strengths**

- The use of the Open-LUCIS framework  (adapted from LUCIS) is well-justified, given its participatory, hierarchical, and suitability-based approach to land-use conflict analysis.
- Clear articulation of the three primary land-use goals (agriculture, human settlement, wildlife conservation) and their sub-objectives ensures methodological transparency.
- The study leverages diverse spatial datasets (e.g., soil properties, DEM, land cover, wildlife habitat suitability) from reputable sources (ISRIC, ESA-Copernicus, WildCRU, etc.), enhancing reproducibility.
- Multi-resolution MGRS grid (250 m and 1000 m) is a pragmatic choice to balance detail and computational efficiency. But I have some concern which is given in the weaknesses and concerns section.
- Weighted Linear Combination (WLC) and Analytical Hierarchy Process (AHP) are appropriately applied for suitability analysis, aligning with established multi-criteria decision-making (MCDM) practices.
- Sensitivity analysis (map removal approach) is a strong addition to assess the robustness of the model, particularly for dynamic human-driven factors.
- The 1–9 to 1–3 reclassification (using natural breaks) and conflict criteria (high/moderate/low) are logically defined and operationalized.

**Weaknesses and Concerns**

- While the study mentions stakeholders, there is no information on:
  - How their inputs were collected (workshops, surveys?) and integrated into the weighting process.
  - Potential biases in stakeholder selection (e.g., overrepresentation of certain groups).
- The weights for suitability analysis (Tables 2–4) are attributed to WildCAT Botswana Trust and domain experts, but:
  - No justification is provided for why specific weights were chosen (e.g., why "Physical" = 0.40 for livestock).
  - Lack of uncertainty quantification (e.g., confidence intervals for weights). Uncertainty Quantification for Weight Justification in Suitability Analysis can be analyzed through expert elicitation with confidence intervals or probabilistic sensitivity analysis or global sensitivity analysis.
- The wildlife habitat suitability layer (from WildCRU) relies on presence-absence data and GLMMs, but:
  - Temporal mismatch: Data years are unclear (e.g., spoor surveys). Are they representative of current conditions?
  - Scale dependency: The 250 m resolution may not capture fine-scale wildlife movements (e.g., elephants). In my opinion, A 250 m resolution grid may not accurately capture elephant habitat needs because it averages large areas into single cells. Elephants use fine-scale features (like 100 m-wide river corridors or small forest patches), but these critical details get "blurred" when combined with surrounding unsuitable land in a 250 m cell. This can lead to errors—either overlooking important habitats (false negatives) or mislabeling fragmented areas as suitable (false positives). Essentially, the model might miss where elephants actually go or overestimate safe zones. I suggest authors to address this.
- The OAT (one-at-a-time) approach is limited; a global sensitivity analysis (e.g., Monte Carlo) would better account for interactions between variables.
- No discussion of how much change in output (e.g., % conflict area shift) was observed after factor removal.
- PyLUSATQ plugin: How was it validated for this study? Is it open-source?
- Land unit generation: The process of creating the composite layer (Figure 5) needs more clarity (e.g., how were MGRS grids merged with settlement layouts?).
- Data preprocessing: Were datasets harmonized to common resolutions/projections?
- The high/moderate/low conflict classification (WL=3 & AG=3, etc.) may oversimplify:
  - No consideration of species-specific conflicts (e.g., elephants vs. lions).
  - No temporal dynamics (e.g., seasonal migration impacts).

3. **Review of Results & Discussion Section**

**Strengths**

- Figures 7a-c and 8a-c effectively visualize land-use preferences, with quantifiable results (e.g., 17% high-agriculture suitability, 41% high-wildlife preference).
- Logical progression from suitability → preference → conflict identification.
- Transparent criteria for conflict classification (low/moderate/high) based on overlapping preferences.
- High-conflict zones align with known wildlife corridors (e.g., commercial farms near Hwange-Chobe corridor), validating the model.
- Table 5 briefly summarizes factor impacts, highlighting trade-offs between conservation and development.
- Emphasizes adaptive land-use planning (e.g., revising zoning maps, habitat restoration) and stakeholder engagement.

**Weaknesses & Concerns**

- No discussion of model validation (e.g., comparison with historical HWC incident data). Are high-conflict zones empirically verified?
- Weighting justification: Crop farming (70%) vs. livestock (30%) weights lack empirical or stakeholder-derived rationale.
- The 250 m resolution (noted in Methods) may misclassify fine-scale conflicts (e.g., elephant crop-raiding at farm edges). Not addressed in Results. Authors have to address this.
- OAT limitations: Ignores factor interactions (e.g., *roads + water proximity* may synergistically increase conflict). A global sensitivity method would strengthen findings.
- Missing climate variables: Droughts or rainfall shifts could alter water-driven conflicts but are excluded.
- Stakeholder input is mentioned but not quantified (e.g., how local knowledge refined weights).

**Final Recommendation**

The methodology is largely sound but requires greater transparency in stakeholder engagement, weight justification, and sensitivity analysis to meet peer-review standards. Authors must address or provide the justifications for the “Weaknesses and concerns” of Introduction, materials and methods as well as results and discussion sections.

**Decision**: **Major Revisions Required**.
