# Supplementary material for: Applying the open-LUCIS framework to identify and characterize human–wildlife conflicts: A case study in Botswana
Source: PLoS One. 2026 Jan 23;21(1):e0336326. doi: 10.1371/journal.pone.0336326 (PMC12829932; doi:10.1371/journal.pone.0336326)
Supplement: Attachment — Submitted filename: Response to Reviewers_PLOS_ONE.docx [file pone.0336326.s004.docx]

Dear Dereje Yazezew Mammo, Ph.D.

On behalf of all co-authors, I would like to express our sincere gratitude to you, the Academic Editor, and the two reviewers for taking the time to thoroughly review our manuscript. We greatly appreciate the comments and constructive suggestions provided, which have significantly contributed to enhancing the quality of our work. In response to the feedback, we have made considerable edits to the manuscript. Specifically, we have revised the introduction, Methods, Results, Discussion, and Conclusion sections to enhance clarity, conciseness, and overall quality. We have provided detailed responses to the reviewer's comments and suggestions below (marked in blue). Also, updates and revisions to the manuscript are marked in green. We believe that these revisions address the concerns raised and contribute to a stronger manuscript. Additionally, we have updated figures to conform to copyright concerns raised.

Thank you once again, and we look forward to hearing from you soon.

Sincerely,

Silas Achidago and Co-authors

**Editor’s Comments**

The manuscript is a useful contribution by way of application of an open-source suitability-based framework for modeling human-wildlife conflicts in Botswana, which is highly appropriate to PLOS ONE's focus on methodological soundness and data-informed research. However, both reviewers also point out several issues that must be revised. Some of the key enhancements include providing explicit details on stakeholder consultations, data collection protocols, and the basis of weight assignments, with appropriate uncertainty estimation or sensitivity analyses. The introduction and discussion should be shortened to focus on the study's specific aims, avoiding general or subjective statements. Descriptions of data preprocessing, model validation, and classification techniques are also absolutely required. These changes, once addressed, will significantly enhance the manuscript's clarity, reproducibility, and scientific impact.

We thank the editor so much for the constructive feedback and for recognizing the contribution of our manuscript. We carefully considered all comments and have revised the manuscript accordingly.

We expanded the methodology to provide explicit information on stakeholder consultations, clarifying how domain knowledge and expert input informed weight assignments and clearly stating the limitations. We also added a subsection describing data sources, collection protocols, and quality assurance measures to improve transparency. The basis of weight assignments was explained more clearly, supported by literature and expert rationale, and the section on uncertainty estimation was strengthened by elaborating on the sensitivity analysis used. This highlighted how robustness and decision-relevance were assessed under data uncertainty. The introduction and discussion were streamlined to focus on the specific aims, results, limitations, and implications of the study while avoiding general or subjective statements. This sharpened the narrative and improved readability.

We are confident that the revisions made are adequate and fully address all concerns raised by the editor and reviewers, and that the manuscript now meets the standards of clarity and methodological rigor required by PLOS One.

Please update your Data Availability statement in the submission form accordingly.

We note that Figures 1,2,3,5,7a,7b,7c,8a,8b,8c and 9 in your submission contain [map/satellite] images which may be copyrighted. All PLOS content is published under the Creative Commons Attribution License (CC BY 4.0), which means that the manuscript, images, and Supporting Information files will be freely available online, and any third party is permitted to access, download, copy, distribute, and use these materials in any way, even commercially, with proper attribution. For these reasons, we cannot publish previously copyrighted maps or satellite images created using proprietary data, such as Google software (Google Maps, Street View, and Earth). For more information, see our copyright guidelines: http://journals.plos.org/plosone/s/licenses-and-copyright.

We require you to either (1) present written permission from the copyright holder to publish these figures specifically under the CC BY 4.0 license, or (2) remove the figures from your submission:

1. You may seek permission from the original copyright holder of Figures 1,2,3,5,7a,7b,7c,8a,8b,8c and 9 to publish the content specifically under the CC BY 4.0 license.

We recommend that you contact the original copyright holder with the Content Permission Form (http://journals.plos.org/plosone/s/file?id=7c09/content-permission-form.pdf) and the following text:

“I request permission for the open-access journal PLOS ONE to publish XXX under the Creative Commons Attribution License (CCAL) CC BY 4.0 (http://creativecommons.org/licenses/by/4.0/). Please be aware that this license allows unrestricted use and distribution, even commercially, by third parties. Please reply and provide explicit written permission to publish XXX under a CC BY license and complete the attached form.”

Please upload the completed Content Permission Form or other proof of granted permissions as an ""Other"" file with your submission.

In the figure caption of the copyrighted figure, please include the following text: “Reprinted from [ref] under a CC BY license, with permission from [name of publisher], original copyright [original copyright year].”

2. If you are unable to obtain permission from the original copyright holder to publish these figures under the CC BY 4.0 license or if the copyright holder’s requirements are incompatible with the CC BY 4.0 license, please either i) remove the figure or ii) supply a replacement figure that complies with the CC BY 4.0 license. Please check copyright information on all replacement figures and update the figure caption with source information. If applicable, please specify in the figure caption text when a figure is similar but not identical to the original image and is therefore for illustrative purposes only.

The following resources for replacing copyrighted map figures may be helpful:

USGS National Map Viewer (public domain): <http://viewer.nationalmap.gov/viewer/>

The Gateway to Astronaut Photography of Earth (public domain): <http://eol.jsc.nasa.gov/sseop/clickmap/>

Maps at the CIA (public domain): <https://www.cia.gov/library/publications/the-world-factbook/index.html> and <https://www.cia.gov/library/publications/cia-maps-publications/index.html>

NASA Earth Observatory (public domain): <http://earthobservatory.nasa.gov/>

Landsat: <http://landsat.visibleearth.nasa.gov/>

USGS EROS (Earth Resources Observatory and Science (EROS) Center) (public domain): [http://eros.usgs.gov/#](http://eros.usgs.gov/)

Natural Earth (public domain): [http://www.naturalearthdata.com/](https://nam10.safelinks.protection.outlook.com/?url=http%3A%2F%2Ftrack.editorialmanager.com%2FCL0%2Fhttp%3A%252F%252Fwww.naturalearthdata.com%252F%2F1%2F010f019850c5392a-01c1a13f-c08e-4615-a888-f100e3014ed3-000000%2FDZ2vg1NNiQ24MGcQRDLX3tQFrQEzPMRWldE7jQlOE5A%3D221&data=05%7C02%7Cs.achidago%40ufl.edu%7Cc2538dcb4c994a55d88f08ddcdcf4e5e%7C0d4da0f84a314d76ace60a62331e1b84%7C0%7C0%7C638893012915388554%7CUnknown%7CTWFpbGZsb3d8eyJFbXB0eU1hcGkiOnRydWUsIlYiOiIwLjAuMDAwMCIsIlAiOiJXaW4zMiIsIkFOIjoiTWFpbCIsIldUIjoyfQ%3D%3D%7C0%7C%7C%7C&sdata=JXilHegkQ3soNK5en%2BboO8tOMQD7hFcs%2Bxb%2BJMbJbzE%3D&reserved=0)

We have replaced all maps that previously contained Google Map backgrounds with OpenStreetMap base maps, which comply with the CC BY 4.0 license requirements. In addition, we have submitted a request for written consent from the original copyright holder, and while we are still awaiting a response, we will promptly update the portal once we receive confirmation.

**Reviewer: 1**

1. While global cases (Kenya, India) are useful, the link to Botswana’s Chobe District (study area) is underdeveloped. A paragraph contrasting global trends with local Chobe dynamics (e.g., elephant conflicts, agricultural expansion) would strengthen focus.

We appreciate this constructive comment, as it’s an important way to strengthen the contextual grounding of our study. In the revised manuscript, we have incorporated this within the first two paragraphs to contrast global trends in human–wildlife conflict with the specific dynamics of Botswana’s Chobe District. (Line 74-90, page 4)

2. Claims LUCIS is "effective" but cites UNDP procurement notices (41,42) as evidence rather than peer-reviewed outcomes. Needs specific examples of LUCIS reducing HWC (e.g., success metrics from Botswana’s Tawana/Chobe).

Thank you for your comment regarding the citations provided for LUCIS. We acknowledge that our original intent was not to claim that the LUCIS framework is already proven to be “effective” in reducing human–wildlife conflict, but rather to highlight its growing relevance in practice. In the revised manuscript, we have addressed this by removing the UNDP procurement notices while situating them within other conservation frameworks and tools to address the identified gap. (Line 84-98, page 4)

3. Open-LUCIS’s limitations (e.g., computational demands, stakeholder bias in weighting) are not addressed.

We appreciate this observation. In the revised manuscript, we have included a discussion of Open-LUCIS’s limitations, specifically highlighting issues such as computational demands and potential stakeholder bias in weighting. These points are now addressed in both the Discussion and concluding sections of the paper. (Line 455-456, page 21 & Line 108-121, Page 21)

4. Lacks discussion of competing land-use frameworks (e.g., Marxan, InVEST) to justify why LUCIS is superior for HWC.

We thank the reviewer for this insightful comment. We agree that it is important to situate LUCIS within the broader context of existing land-use planning and conservation frameworks. In the revised manuscript, we have incorporated a discussion comparing LUCIS with other widely used approaches, such as Marxan, InVEST, and Maxent. We have therefore clarified that the strength of LUCIS lies in its explicit focus on conflict identification and suitability-based land allocation, which makes it particularly well-suited for assessing human–wildlife conflict scenarios. (Line 87-90, page 4)

5. No mention of spatial ecology theories (e.g., landscape permeability, source-sink dynamics) that ground habitat-suitability modeling.

We thank the reviewer for this thoughtful suggestion. We acknowledge the importance of spatial ecology theories such as landscape permeability and source–sink dynamics in grounding habitat-suitability modeling. However, the primary focus of our study is on the application of the Open-LUCIS framework to analyze land-use dynamics and identify potential areas of human–wildlife conflict, rather than delving deeply into ecological theories. For this reason, we adopted a habitat-suitability layer as a practical means of integrating ecological considerations with wildlife habitat needs, without delving into the broader ecological underpinnings. We have clarified this focus in the revised manuscript to drive home this point.

6. Mentions "domain knowledge" and "local priorities" but doesn’t detail how stakeholders were involved (e.g., workshops, surveys) or how their inputs were weighted.

While the study mentions stakeholders, there is no information on:

♣ How their inputs were collected (workshops, surveys?) and integrated into the weighting process.
♣ Potential biases in stakeholder selection (e.g., overrepresentation of certain groups).

We appreciate this important comment. In the revised manuscript, we have clarified that domain knowledge was obtained through meetings and discussions with the WildCAT team, which provided insights to guide the operationalization of the LUCIS framework for demonstration purposes. We acknowledge that this approach does not constitute a formal stakeholder engagement process (e.g., workshops or surveys) and therefore carries the potential for bias. This limitation, along with others, has been explicitly noted in the manuscript to ensure transparency regarding the scope and constraints of the study.

7. The weights for suitability analysis (Tables 2–4) are attributed to WildCAT Botswana Trust and domain experts, but:
   ♣ No justification is provided for why specific weights were chosen (e.g., why "Physical" = 0.40 for livestock).

♣ Lack of uncertainty quantification (e.g., confidence intervals for weights). Uncertainty Quantification for Weight Justification in Suitability Analysis can be analyzed through expert elicitation with confidence intervals or probabilistic sensitivity analysis or global sensitivity analysis.

The wildlife habitat suitability layer (from WildCRU) relies on presence-absence data and GLMMs, but:
♣ Temporal mismatch: Data years are unclear (e.g., spoor surveys). Are they representative of current conditions?

We acknowledge that the justification for the specific weight values was not fully elaborated in the manuscript. We have revised the text to provide a clearer explanation of how these values were determined.

Regarding uncertainty quantification, we agree that methods such as expert elicitation with confidence intervals or probabilistic sensitivity analysis would significantly strengthen the confidence level of the assigned weights. However, in this study, the domain and expert knowledge were obtained from a single source (WildCAT), which limited our ability to conduct such an exercise across multiple expert opinions to derive confidence intervals. We nonetheless recognize the value of these approaches and mention them as limitations of this study. (Line 451-458, page 21)

8. Scale dependency: The 250 m resolution may not capture fine-scale wildlife movements (e.g., elephants). In my opinion, A 250 m resolution grid may not accurately capture elephant habitat needs because it averages large areas into single cells. Elephants use fine-scale features (like 100 m-wide river corridors or small forest patches), but these critical details get "blurred" when combined with surrounding unsuitable land in a 250 m cell. This can lead to errors—either overlooking important habitats (false negatives) or mislabeling fragmented areas as suitable (false positives). Essentially, the model might miss where elephants actually go or overestimate safe zones. I suggest authors to address this.

We sincerely appreciate the reviewer’s detailed observations regarding the wildlife habitat suitability layer derived from WildCRU. We acknowledge that these are important considerations that could indeed influence the accuracy of habitat modeling, particularly for species such as elephants with fine-scale movement patterns. However, we would like to clarify that the wildlife habitat suitability layer was not generated as part of this study but was instead incorporated from WildCRU’s existing work. As such, while the concerns raised are valid, we are unable to directly address them here. Nonetheless, we agree that these limitations are important to highlight and have been captured in the limitations of the paper. (Line 451-458, page 21)

9. The OAT (one-at-a-time) approach is limited; a global sensitivity analysis (e.g., Monte Carlo) would better account for interactions between variables.

The OAT sensitivity analysis was applied in line with the exploratory nature of this study, which is intended as a methodological demonstration rather than a decision-support tool. OAT offers a clear and transparent approach that facilitates the isolation of individual factors, making it straightforward to trace how each contributes to variations in conflict outcomes while holding other factors constant.

10. No discussion of how much change in output (e.g., % conflict area shift) was observed after factor removal.

We thank the reviewer for this observation. In the revised manuscript, we have included a discussion of the magnitude of change in model outputs, highlighting the percentage shifts in conflict areas following the removal of individual factors. We have also updated Table 5 to include these percentage changes.

11. PyLUSATQ plugin: How was it validated for this study? Is it open-source?

We appreciate the reviewer’s query. The PyLUSAT-QGIS plugin is indeed an open-source tool, developed to extend the PyLUSAT library functionalities within QGIS. Its development and validation are documented in a peer-reviewed publication, which we cited in the manuscript.

“*Chen C, Judge J, Hulse D. PyLUSAT: An open-source Python toolkit for GIS-based land use suitability analysis. Environ Model Softw [Internet]. 2022 May [cited 2024 Jan 15];151:105362.Availablefrom: https://linkinghub.elsevier.com/retrieve/pii/S1364815222000688*”

12. Land unit generation: The process of creating the composite layer (Figure 5) needs more clarity (e.g., how were MGRS grids merged with settlement layouts?).

We thank the reviewer for pointing out the need for greater clarity. In the revised manuscript, we have specified that the land unit composite layer was created by merging the settlement layout and MGRS grid vector layers using the merge vector geoprocessing tool. This clarification has been incorporated into the revised manuscript. (Line 196-201, page 9)

13. Data preprocessing: Were datasets harmonized to common resolutions/projections?

We appreciate the reviewer for pointing this out. In the revised manuscript, we have added a sentence in the data sources section clarifying that all datasets were standardized to a common spatial resolution and projection (EPSG:32734 – WGS 84 / UTM Zone 34S) to ensure consistency and accuracy across analyses. (Line 154-156, page 7)

14. The high/moderate/low conflict classification (WL=3 & AG=3, etc.) may oversimplify:
    ♣ No consideration of species-specific conflicts (e.g., elephants vs. lions).
    ♣ No temporal dynamics (e.g., seasonal migration impacts).

We thank the reviewer for this insightful observation. We agree that the high/moderate/low conflict classification may represent an oversimplification. Our intent was to provide a broad, landscape-level guide for identifying potential hotspots of human–wildlife conflict, rather than a species-specific or temporally dynamic assessment. Given the static nature of the available data, our analysis was constrained to a generalized framework, and we have explicitly acknowledged these limitations in the revised manuscript. We also note that future studies should incorporate species-specific conflict patterns (e.g., elephants versus carnivores) and temporal factors such as seasonal migrations and resource availability to provide a more detailed and ecologically nuanced understanding of conflict dynamics.

15. No discussion of model validation (e.g., comparison with historical HWC incident data). Are high-conflict zones empirically verified?

We thank the reviewer for this important comment. While a model validation was not conducted, we referenced Figure 3 in the manuscript, which presents reported human–wildlife incidents in Pandamatenga between 2007 and 2023 from the Problem Animal Control unit of the Department of Wildlife and National Parks, Botswana. These records show a reasonable alignment with the identified conflict-prone hotspots in our analysis, particularly around the commercial farming areas.

16. Weighting justification: Crop farming (70%) vs. livestock (30%) weights lack empirical or stakeholder-derived rationale.

We appreciate the reviewer’s observation. The crop farming (70%) versus livestock rearing (30%) weighting was guided by domain knowledge obtained through discussions with the WildCAT team as an indication of the crop farming being more dominant than livestock rearing. We acknowledge that these weights were not derived from an empirical or stakeholder-based process, and the intent was not to present them as definitive values but rather as illustrative inputs for demonstrating the framework’s application. This clarification has been added to the revised manuscript. (Line 214—223, page 10)

17. The 250 m resolution (noted in Methods) may misclassify fine-scale conflicts (e.g., elephant crop-raiding at farm edges). Not addressed in Results. Authors have to address this.

We appreciate and agree with the reviewer’s concern, and we acknowledge that the use of 250 m resolution data may misclassify fine-scale conflicts such as elephant crop-raiding along farm edges (as indicated above). This is a valid point and has been incorporated as a limitation in the revised manuscript. As noted, the study relied on readily available datasets to demonstrate the application of the LUCIS framework, and the results should therefore be viewed as indicative rather than absolute.

18. OAT limitations: Ignores factor interactions (e.g., roads + water proximity may synergistically increase conflict). A global sensitivity method would strengthen findings.

We thank the reviewer for this valuable suggestion. We agree that one-at-a-time (OAT) sensitivity analysis does not capture potential interactions among factors (e.g., roads and water proximity synergistically influencing conflict) and that a global sensitivity method would provide a broader and more comprehensive perspective. This limitation has been noted in the revised manuscript, and we have suggested that future research incorporate global sensitivity approaches. As this paper primarily serves to demonstrate the application of the LUCIS framework, OAT was used for its practical and transparent approach.

19. Missing climate variables: Droughts or rainfall shifts could alter water-driven conflicts, but are excluded.

We agree with the reviewer’s observation and acknowledge that incorporating climate variables such as droughts or rainfall variability would greatly enhance the relevance of the research by capturing additional drivers of water-related conflict. This limitation has been noted in the revised manuscript, with a recommendation that future studies integrate climate dynamics to strengthen the analysis. (Line 451-458, page 21)

20. Stakeholder input is mentioned but not quantified (e.g., how local knowledge refined weights).

We appreciate this observation. In the revised manuscript, we have clarified that there was no direct stakeholder input into the weighting process. Instead, domain knowledge was obtained through discussions with the WildCRU team, who work directly with stakeholders in the study area. This distinction has now been made explicit to avoid any ambiguity regarding the source and nature of the knowledge used. (Line 108-121, page 5)

21. Final Recommendation: The methodology is largely sound but requires greater transparency in stakeholder engagement, weight justification, and sensitivity analysis to meet peer-review standards. Authors must address or provide the justifications for the “Weaknesses and concerns” of the Introduction, materials and methods, as well as results and discussion sections.

We sincerely thank the reviewer for this comprehensive recommendation. In the revised manuscript, we have strengthened the transparency of our methodology by clarifying the role of domain knowledge in place of direct stakeholder engagement, explaining how weights were assigned for demonstration purposes, and elaborating on the limitations of the one-at-a-time sensitivity analysis. We have also expanded the Discussion and Conclusion to explicitly acknowledge these weaknesses and outline how future studies can address them through stakeholder-driven weighting, empirical validation, and the use of global sensitivity methods. These revisions aim to ensure that the methodological choices and their implications are presented with greater clarity and transparency in line with peer-review standards.

**Reviewer: 2**

1. The introduction is lengthy and reads more like a general review of human-wildlife conflict (HWC) and other topics rather than a concise introduction to their study. As a result, the lead-up to the description of study goals is less clear. The authors include details and examples from numerous studies that are minimally relevant to this study. The authors also insert numerous subjective statements about what should be done (e.g., line 127: “To effectively manage HWC, an integrated…approach…is required.” ). It would be helpful to describe the spatial extent and temporal scale in the goals section. The introduction of the LUCIS framework also comes out of nowhere (and it is not entirely clear at first this is for the Chobe District). The paragraph describing generically what is in the methods section and results can be deleted.

We thank the reviewer for these constructive observations on the introduction. The concerns raised about length, generality, and clarity have been addressed. The introduction has been streamlined to focus more directly on the scope and objectives of the study, with extraneous details and minimally relevant examples removed. Subjective statements have been revised to maintain a neutral and scientific tone. The study goals are now presented with clearer context and the introduction of the LUCIS framework is framed more explicitly in relation to its application in the study area.

2. The methods are generally informative, but some details could be described in greater detail (e.g., not all readers will know about “the” WildCRU (e.g., simply explain its relationship to Oxford), and it is not clear what the “domain knowledge” from WildCRU entails. The study area description probably includes more detail than is needed for this study.

We thank the reviewer for this observation. A description of WildCRU, including its affiliation with the University of Oxford, has been added to clarify its role. The explanation of “domain knowledge” has also been expanded to better illustrate its contribution to the study. Additionally, the study area description has been streamlined to remain focused on details directly relevant to the analysis. (Line 108-121, page 5)

3. How or why did the study identify agriculture, human settlement, and wildlife as the primary LUP goals (line 260)? On page 13 the authors state that the weights were based on “domain knowledge and expert opinion” from the WildCAT Botswana Trust, but the authors do not explain how this was derived and cite an unpublished report as the source.

We appreciate this comment and have clarified our rationale in the methodology section. Agriculture, human settlement, and wildlife were identified as the dominant and competing land-use objectives in the study area based on domain knowledge. This was informed through a series of discussions with the WildCAT team via online meetings and complemented by contextual insights from a field visit to Botswana. We also note that the citation in question referred specifically to the Wildlife Habitat Suitability study we adopted, rather than to the derivation of weights in this paper. These have been clarified in the revised manuscript.

4. The results are interesting but largely a function of the weights applied in the model. Habitat suitability models in general suffer from a risk of subjective weighting. For example, the authors describe and “objective weight” (table 2) of 0.4 for physical, 0.3 economic, and 0.3 for wildlife for livestock (and do this again for crop farming and human development). These were further subdivided by “sub-objective weights”. The results of the model would be very different if different weights were selected, and there was no effort to provide a range of weights, or variances, that could provide a range of possible outcomes.
   The results also include some management goals that seem at odds with research questions (e.g., line 341: “…we aimed to concentrate the growth of human settlement to enhance shared existing infrastructure and resources.” Why? This seems like an important a priori decision).

We acknowledge the reviewer’s concern regarding the role of weighting in shaping model outcomes. As noted, the results are indeed sensitive to the weights applied, and this study did not attempt to test alternative weighting schemes. Rather, the weights were derived from domain knowledge and were intended to serve as a demonstration of how the LUCIS framework can operationalize land-use objectives into spatially explicit outputs. We have clarified this in the manuscript, emphasizing that the analysis is exploratory and methodological, not prescriptive. The management-oriented goals (e.g., concentrating settlement growth near existing infrastructure) are not recommendations but derived from WildCAT and serve as an example of how planning objectives can be structured within the framework. We also note this limitation in the discussion, highlighting the potential for future work to explore alternative weights, variance testing, and sensitivity to different planning scenarios.

5. The authors use a Jenks classification to categorize the suitability layers. This classification is typically used to maximize color separation on maps—but it can lead to extremely varied classes to achieve that goal. For example, one class could include an extremely large range while another class could include an extremely small range, depending on the histogram. How is this related to “preference”? The authors may want to further clarify why this provides (line 381) “…optimal and significant class intervals.”

We thank the reviewer for the comment. The use of the Jenks classification system was motivated by its strength in optimizing class breaks. Specifically, Jenks minimizes within-class variance while maximizing between-class variance, thereby producing groupings that best reflect the natural structure of the data. While it is true that class ranges can vary in size depending on the underlying distribution, this variation is a product of the method’s optimization process, which ensures that each class is internally coherent and statistically distinct from others. In this study, the resulting classes are interpreted as “preferences” in the sense that they represent modeled suitability outcomes that most clearly separate areas of high, moderate, and low potential within the LUCIS framework. We have clarified this rationale in the revised manuscript to highlight why the Jenks method provides optimal and meaningful class intervals for our analysis. (Line 292-296, page 14)

6. The manuscript is generally well written and edited. However, the manuscript includes some very lengthy paragraphs (e.g., paragraph starting p 5 line 113 and ending page 7 line 148!) that are difficult to read and could be shortened into focused and concise paragraphs.

We appreciate the reviewer’s comment on this. The lengthy paragraphs identified have been revised and shortened into more focused and concise sections, as well as other aspects of the manuscript, to improve readability and flow.

7. The authors carried out a sensitivity analysis but do not include validation steps to help us understand whether the weights they selected are relevant in the real world. Understanding which variables impact the model more or less (the sensitivity) is helpful, but without some kind of validation steps it is just a hypothetical model. What would happen if different weights were used? At the very least, the authors should discuss the limitations of this type of subjective HSI approach

We thank the reviewer for this observation. The OAT sensitivity analysis was applied in line with the exploratory nature of this study, which is intended as a methodological demonstration rather than a decision-support tool. OAT provides a transparent means of isolating individual factors, making it straightforward to trace how each contributes to variation in conflict outcomes while holding other factors constant. We have also explicitly acknowledged the absence of validation as a limitation in the revised discussion and proposed its inclusion in a more comprehensive study. (Line 323-327, page 15 & 453-455, Page 21)

8. The authors provide a series of specific “preferences” (e.g., no substantial overlap in land use preferences between people and animals in 70% of the examined region…”), but how much of this is simply a function of separating conserved wildlands from human settlements and agricultural/livestock grazing lands? What does it mean that “41% of the region…is preferred by wildlife.”? Does wildlife really “prefer” just some of this land, or is this simply a function of their model parameters?

We thank the reviewer for this helpful comment. We agree that the percentage of overlap is largely a function of the weights applied to the objectives. However, this separation is not arbitrary; it reflects outcomes of land-use planning processes shaped by competing interests, with each land use seeking to optimize its objectives. With respect to the statement that “41% of the region is preferred by wildlife,” we acknowledge that wildlife does not literally “prefer” land. Rather, this terminology reflects suitability modeling that identifies areas meeting the ecological conditions necessary to support wildlife habitat (e.g., forage, water, and cover). To prevent misinterpretation, we have revised the manuscript to clarify that “preference” refers to modeled suitability outcomes within the LUCIS framework, not to species choice or behavior. (Line 357-359, page 16)

“*Here, “preference” does not denote literal selection by people or wildlife, but rather the modeled outcome of weights applied to the objectives within the LUCIS framework, offering a basis for identifying potential areas of conflict*.”

9. The discussion, like the introduction is lengthy and includes statements that are unnecessary (e.g., line 529: “This study fundamentally addresses the emergence of HWC….” Really? Or line 531: “The results of this study are particularly crucial for land administrators…” says who?). The authors would benefit from a narrower and more focused discussion of their results, the strengths and limitations of their methods, future directions, and the narrower set of management implications that derive from their results rather than repeating statements about how crucial their results are.

We appreciate this valuable feedback. The introduction and discussion have been substantially revised to be more focused and concise, with unnecessary or subjective statements removed. The revised version now emphasizes the results, strengths, and limitations of the methods, and clear directions for future research, while presenting a narrower set of implications derived directly from the findings.

10. The conclusion section is unnecessary and is largely repetitive. Anything novel should be included in the discussion. I would encourage the authors to: shorten and focus the introduction and discussion, remove subjective “should statements” from the introduction and provide concise recommendations relevant to their study results in the discussion, the description of how the weights were developed needs more clarification in the methods section, and the discussion would benefit from a description of possible limitations (including why there was no validation step). The title of the paper might be more informative if it was something like “applying the LUCIS framework to HWC in Botswana” or something similar.

We thank the reviewer for these thoughtful and constructive comments. All points raised have been carefully addressed throughout the revised manuscript. Specifically, the introduction has been streamlined to focus more directly on the paper's scope and objectives. The discussion and conclusion have been merged and refined to present a more concise and integrated narrative, avoiding unnecessary repetition while highlighting the key findings and implications. The description of weights has been clarified in the methods section, noting that this study is presented as a test case to demonstrate the application of the LUCIS framework rather than as a finalized decision-support tool. Finally, the title of the paper has been revised to more accurately reflect the work's objective and its emphasis on applying the LUCIS framework to human–wildlife conflict in Botswana.

“Applying the Open-LUCIS Framework to Identify and Characterize Human–Wildlife Conflicts: A Case Study in Botswana”
