# Supplementary material for: Applying the open-LUCIS framework to identify and characterize human–wildlife conflicts: A case study in Botswana
Source: PLoS One. 2026 Jan 23;21(1):e0336326. doi: 10.1371/journal.pone.0336326 (PMC12829932; doi:10.1371/journal.pone.0336326)
Supplement: Attachment — Submitted filename: PONE-D-25-30977_R1 - Reviewer response.docx [file pone.0336326.s003.docx]

**Reviewer response: Detailed Point-by-Point Assessment:**

**1. & 2. Introduction & Justification of LUCIS:**

**Adequately Addressed.** The introduction is now more focused and provides a stronger, better-justified rationale for using LUCIS in this specific context.

**3. & 6. & 20. & 21. Stakeholder Engagement and Limitations:**

**Well Addressed.** The manuscript is now much more honest and transparent about its methodological boundaries. The distinction between expert domain knowledge and formal stakeholder engagement is crucial and has been clearly made.

**4. Discussion of Competing Frameworks:**

**Fully Addressed.** This addition effectively justifies the choice of the LUCIS framework for an HWC study.

**5. Spatial Ecology Theories:**

**Partially Addressed.** While a deeper theoretical discussion would have strengthened the ecological foundation, the reviewer can accept the authors' rationale that it falls outside the paper's primary scope as a methodological demonstration. The response is reasonable.

**7. & 16. Weight Justification and Uncertainty:**

**Adequately Addressed.** The justifications are more explicit, and the critical limitation regarding uncertainty is now openly discussed, which is a significant improvement.

**8. & 17. Data Resolution (250m):**

**Adequately Addressed.** It is fair to state this as a limitation of using existing data. The concern has been acknowledged and documented for the reader.

**9. & 10. & 18. Sensitivity Analysis:**

**Substantially Addressed.** The addition of quantitative change metrics greatly strengthens the sensitivity analysis. While a global method would be superior, the revised analysis is now far more informative and meets a minimum standard for a methodological paper.

**11. PyLUSATQ Validation:**

**Fully Addressed.**

**12. & 13. Methodological Clarity:**

**Fully Addressed.** The methods are now more reproducible.

**14. & 15. Model Oversimplification and Validation:**

**Adequately Addressed.** Given the paper's aim as a framework demonstration, openly stating these simplifications is an appropriate response.

**19. Climate Variables:**

**Adequately Addressed.**

**Overall Conclusion for Reviewer:**

The authors have been highly responsive. The revised manuscript is markedly improved in terms of methodological transparency, acknowledgment of limitations, and clarity of justification.

**Strengths of the Revision:** The explicit discussion of stakeholder input limitations, the improved rationale for weight assignments, the quantitative enhancement of the sensitivity analysis, and the thorough listing of study limitations are particularly commendable.

**Remaining Minor Objections:** A purist might still argue for a more robust sensitivity analysis or a deeper ecological grounding, but the authors have provided reasonable justifications for their choices within the scope of this demonstration paper.

**Final Recommendation:** The authors have adequately addressed all my major concerns. The manuscript now meets the expected standards for methodological rigor and transparency for publication in PLOS ONE. **I would recommend acceptance**.
